# Incidence of suicide among teenagers and young adults in Transkei, South Africa

**DOI:** 10.4102/phcfm.v1i1.45

**Published:** 2009-06-30

**Authors:** Banwari L. Meel

**Affiliations:** 1 Department of Forensic Medicine, Walter Sisulu University, South Africa

**Keywords:** hanging, suicide, HIV/ AIDS, unnatural deaths, autopsy

## Abstract

**Background:**

Transkei is the least developed of the former black homelands in South Africa and has a population of about 4 million. People in this area are poor and depend mainly on the income from migratory workers to the gold mines. Suicide is a complex problem, with no definitive causative agent that has been identified as yet. Suicide among teenagers and young adults is now emerging as an important mental health issue. Suicidal behaviour in the population is under-researched, and therefore under-reported.

**Method:**

This is a retrospective record review from 1993 to 2003, carried out in the Umtata General Hospital mortuary. About 1 000 medico-legal autopsies are conducted annually, and the mortuary caters for a population of about 400 000.

**Results:**

Of the 10 340 medico-legal autopsies, 398 (3.84%) suicide cases were due to hanging. The number has increased from 5.2 per 100 000 of the population in 1993 to 16.2 in 2003. More than a half (55%) of the hangings were of people less than 30 years of age, and less than one-quarter (23%) of these victims were younger than 20 years. The rate in males has increased from 4.5 (1993) to 14 per 100 000, and in females from 0.7 to 2.2 per 100 000. The male/female ratio is recorded highest (9 : 1) in the 20- and 29-year age group.

**Conclusion:**

There is an increasing incidence of suicides among young adults. Suicidal tendency among teenagers and young adults is emerging as an important health issue that needs to be addressed.

## INTRODUCTION

According to World Health Organization (WHO) estimates, and based on current trends, by the year 2020 approximately 1.53 million people will die from suicide, and 10 to 20 times more people will attempt suicide worldwide. This represents an average of one death every twenty seconds and one attempt every one to two seconds.^1^ Between 1950 and 1995 there was an increase of 49% in male suicides and 33% in females.^1^ Suicide is a major cause of death in the young.^2, 3^ Official suicide statistics, which have proven to be underestimates, nevertheless show that up to 20% of male and 28% of female deaths among adolescents in the industrialised world are caused by suicide. In the adolescents, suicide ranks among the first three causes of death.^4^

In South Africa in 1990, the overall suicide rate was 17.2 per 100 000, which is slightly higher than in the WHO report.^5^ Initial estimates from the South African National Burden of Disease Study (2000) indicated suicide as the tenth of 20 leading causes of mortality.^6^ A study carried out by the author in the Transkei area in 2003 showed that there is an increase in the incidence of deaths due to hanging. Nearly two-thirds of these victims were teenagers and young adults.^7^ Suicidal ideation and plans to commit suicide are highest among Asians, closely followed by white pupils and lowest among black pupils.^8^ Black youth do not usually consider suicide as an option when they cannot cope. However, they do so when in severe depressive moods.^9^

Poor school performance may predispose children to hopelessness and depression, which are often thought to be essential components of suicide.^10^ It has been well documented that depression shows a strong association with suicide.^11^ Depression and hopelessness are generally regarded as the essential ingredients of completed suicide.^12^ Over 90% of suicide victims have a psychiatric disorder at the time of death.^13^ However, most psychiatric patients do not commit suicide.^14^

Suicide rates in the young are positively related to the proportions of adolescents in the population,^15^ and increases with unemployment and alcohol use.^16^ South Africans consume well over 6 billion litres of alcoholic beverages a year. A high level of alcohol misuse has been reported among residents of disadvantaged communities.^17^ A study in 2003 carried out by the author in Transkei showed that financial hardships were the reason for 87% of suicides.^18^ However, these observations only partially describe the phenomenon of suicide at an aggregate level. They do not provide us with an individual psychological understanding of why that person killed him- or herself, which is so necessary for suicide prevention.^19^

There are abundant political and health discussions yet only a few studies on suicide among teenagers and young adults. The recent increase in suicide deaths among adolescents and young adults has prompted researchers to identify risk factors that may be clinically relevant and contribute to public health preventive efforts.^20^ The purpose of this study is to highlight the problem of suicide among teenagers and young adults in the Transkei region.

## METHOD

The Umtata (Mthatha) General Hospital (UGH) mortuary deals with about 1 000 medico-legal autopsies (of unnatural deaths) a year from the Mthatha area, which has a population of about 400 000. It is the teaching hospital of the Walter Sisulu University Medical School. The objective of this study was to determine the incidence of suicide (specifically by hanging) among teenagers and young adults in the Transkei region.

This is a record review of deaths due to hanging during the period January 1993 and December 2003. All medico-legal autopsies (10 340) were recorded in a register at the mortuary. The mortuary is located on the hospital premises. All deaths from unnatural causes in the region are notifiable to the police, who then request medico-legal autopsies. A medico-legal autopsy is conducted, usually at the request of police, after an unnatural death in which foul play is suspected.

The word ‘suicide’ has been used synonymously with hanging. No homicidal hangings were reported during this period. Hence, all cases were considered suicides. The names, addresses, age and causes of deaths have been recorded in the register. All autopsy records were reviewed and analysed manually. This increased the reliability and validity of the study. The results were compiled and analysed by means of the Epi-info 6.4 computer program.

## RESULTS

**FIGURE 1:**
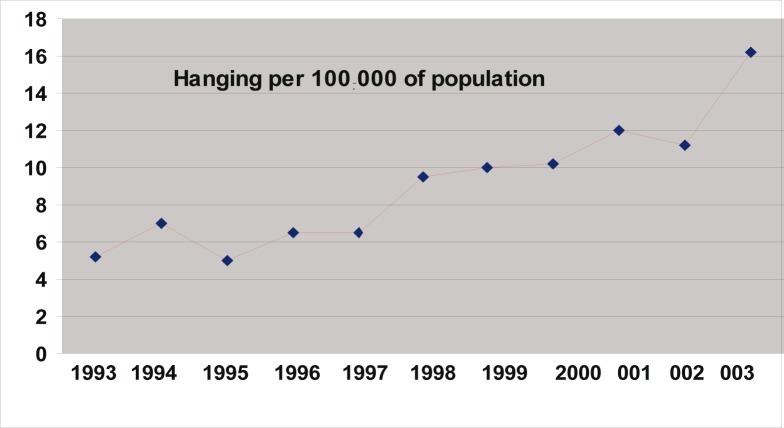
Trends in suicides in the Transkei region of South Africa (1993–2003)

**FIGURE 2:**
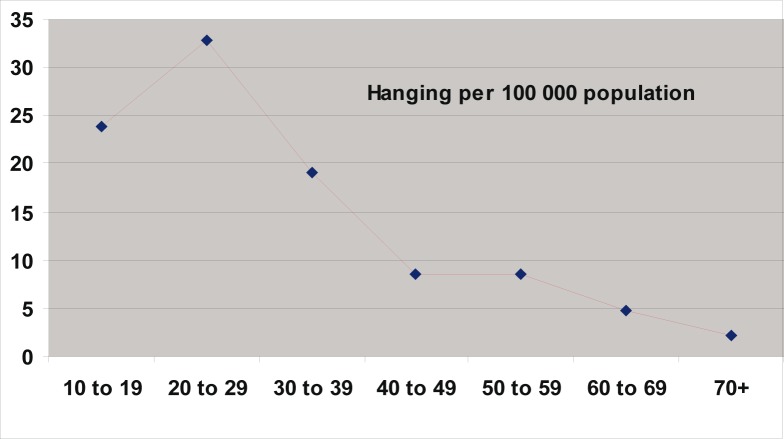
Suicides in different age groups in the Transkei region of South Africa (1993–2003)

**FIGURE 3:**
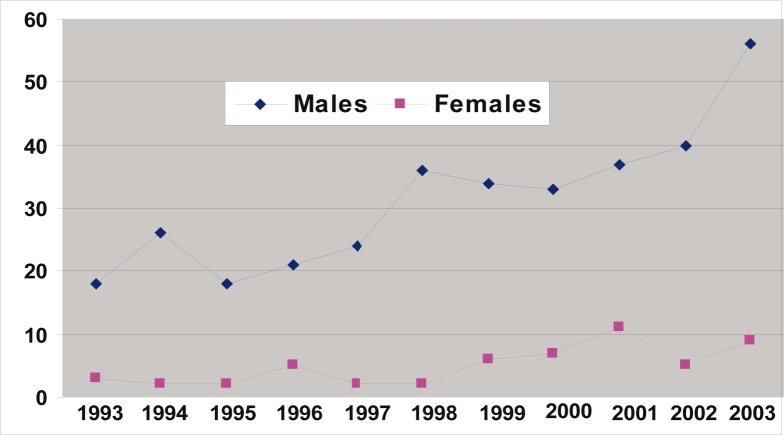
Trends of suicides among both genders in the region of Transkei of South Africa (1993–2003)

**FIGURE 4:**
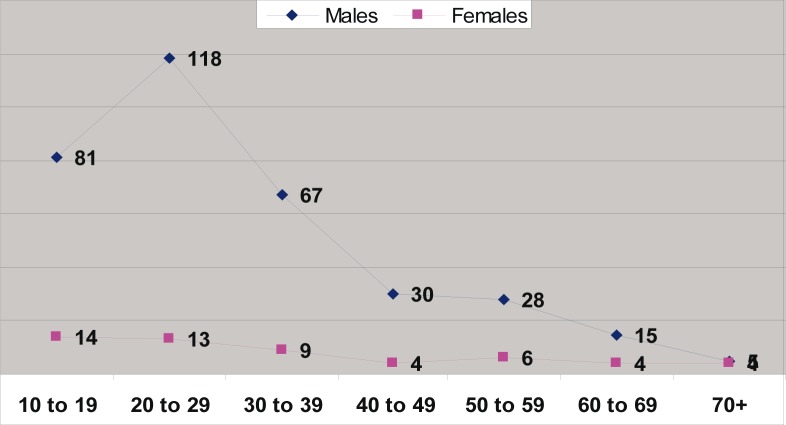
Suicides in different age groups and in both genders in the Transkei region of South Africa (1993–2003)

**FIGURE 5:**
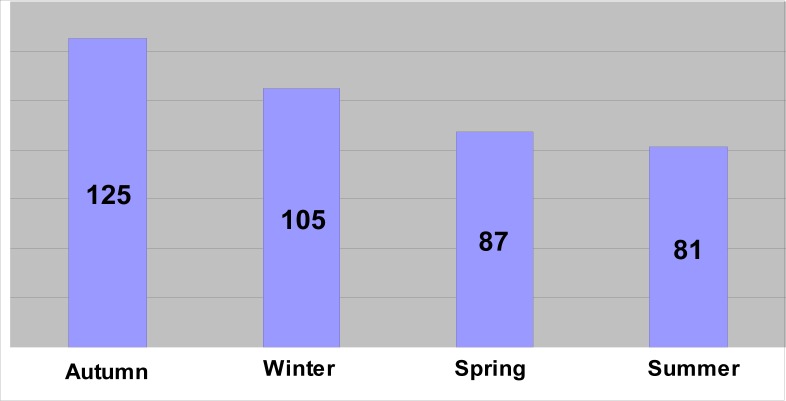
Suicides in different seasons of the year in the Transkei region of South Africa (1993–2003)

**FIGURE 6:**
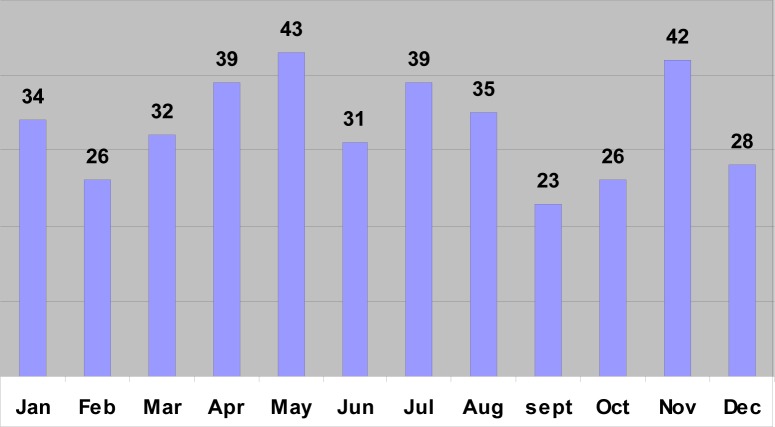
Suicides in the different months of the year (1993–2003)

Of the 10 340 medico-legal autopsies, 398 (3.84%) were related to hangings in all the age groups. The number increased from 5.2 per 100 000 of the population in 1993 to 16.2 in 2003 (Table 1 and Figure 1). More than half (55%) of the hanging victims were younger than 30 years old, and almost one-quarter (23%) were younger than 20 years old (Table 2 and Figure 2). The rise in the rate in males was from 4.5 to 14 per 100 000, and in females from 0.7 to 2.2 (Table 1 and Figure 3). The male/female ratio is recorded highest (9:1) in the 20 to 29-year age group and lowest (1.2:1) in those above 70 years of age (Table 2 and Figure 4). The peak is in the age group 20 to 29 years (Figure 4). There is no consistency in the seasonal and monthly variation of suicides (Figures 5 and 6).

**TABLE 1:** Hanging in Transkei (1993-2003)

YEARS	SUICIDE PER 10 000 POPULATION		TOTAL

	MALES	FEMALES	
1993	4.5	0.7	5.2
1994	6.5	0 5	7
1995	4.5	0.5	5
1996	5.2	1.2	6.4
**1997**	6	0.5	6.5
1998	9	0.5	9.5
1999	8.5	1.2	9.7
2000	8.2	2	8.4
2001	9.2	2.7	11.9
2002	10	1.2	11.2
**2003**	14	2.2	16.2

**TABLE 2:** Suicide: age group vs gender between 1993 and 2003 (n = 398)

AGE GROUPS	%SUICIDE	% GENDER		RATIO

		MALE (N = 344)	FEMALE (N = 54)	
10-19	23	81 (20.2%)	14 (3.6%)	5.8
20-29	32	118 (29.7%)	13 (3.2%)	9
30-39	19	67 (16.8%)	9 (2.3%)	7.4
40-49	8.5	30 (7.6%)	4(1%)	7.5
50-59	8.5	28 (7%)	6(1.5%)	4.7
60-69	4.8	15(3.7%)	4(1%)	3.7
70+yrs	2.2	5(1.2%)	4 (1%)	1.2

## DISCUSSION

This retrospective study is one of the few inquiries into suicides in the Xhosa community in Transkei. There are only a few published articles on the issue of suicides. Most of the people in this area considered suicide as a cowardly act, which is generally not accepted in the Xhosa community. An individual can commit suicide due to social causes.^21^ The rate of suicide varies in different geographical areas within countries, and among different tribes. Suicide among individuals in society was dependent on the person's level of social integration. The lower the integration, the higher the tendency to commit suicide.^22^ This study has shown that there was an increase in the rate of hangings from 5.2 per 100 000 (1993) to 16.2 per 100 000 (2003) in all age groups (Table 1 and Figure 1). In 2001, 2 500 suicides were reported by NIMSS in South Africa. Hanging was the method in 42.3%, firearms in 29.4% and poisoning in the rest of the cases among all age groups.^23^ Based on these facts, one can assume that the total suicides in this region should be 13 per 100 000 (1993) and 41 per 100 000 (2003) respectively in all age groups. This is almost two and a half times higher than the WHO average (17 per 100 000) in the same age group.^1^ A recent (2006) study carried out showed that the overall age-standardised suicide rates for six cities was 25.3/100 000 for men and 5.6/100 000 for women.^24^ Mthatha is situated in the rural part of South Africa. It is generally presumed that suicides occur more in urban areas, but this is not necessarily true. Rural populations have less access to, and fewer, resources than urban populations. They are much poorer and weaker, and at higher risk of suicide because they are confronted more frequently with hazardous situations on a daily basis. Close to three-quarters (74%) of the province's population earn less than R1 500 per month and 41% of households have a monthly income under R500 a month. The Eastern Cape has the country's second highest proportion of poor (44.5%), with the equivalent figure in the Transkei no less than 92%.^25^

**FIGURE 7:**
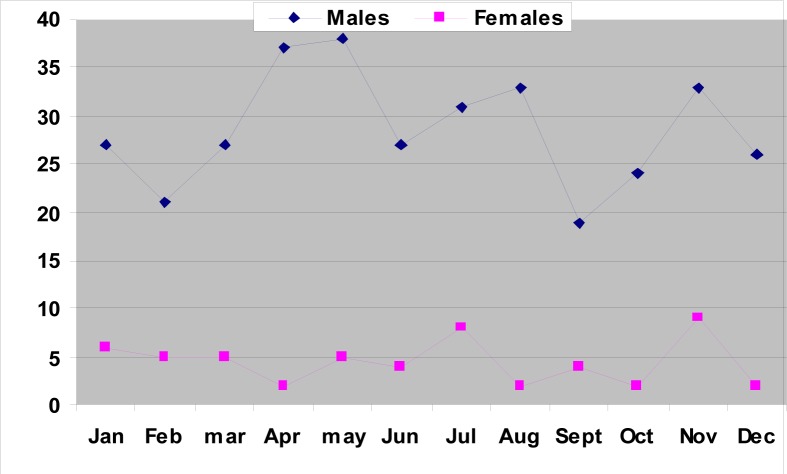
Suicides in relation to months in both gender in the Transkei region of South Africa (1993–2003)

During the past five decades, suicide rates have been increasing globally by approximately one and a half times, i.e. from 10 (1950) to 16 per 100 000 (1995), but in Mthatha it has increased by three times a decade. If the South African suicide rate continues as it is, by 2013 it will be the highest in the world. There has been an incremental increase in hangings from 1993 to 2003. It has increased from 5.2 per 100 000 population (1993) to 7 per 100 000 population (1994), and keeps increasing every year. This pattern appeared in a graph like a ‘ladder pattern’ (Figure 1). Risk factors that appear to be universal include youth, low socioeconomic standing, substance use and previous suicide attempts.^26^ A study conducted by the author showed that common factors such as poverty, unemployment and alcohol consumption are prevalent in this region.^18^ The unemployment rate has increased from 36% in 1996 to 43% in 2001, and poverty from 34% to 39% during the same period in the Eastern Cape.^27^ The only other contributing factor, which has been added since 1996, is the epidemic of HIV/AIDS. Early studies suggest that suicide risk is 20 to 36 times higher in HIV-positive people than in the general population. Recent trends in America, however, show a decline. This is not true in Africa, including the Eastern Cape province in South Africa.^28^ It is not known how many suicides are the result of HIV/AIDS, as both carry stigma and lead to discrimination. A study carried out in 2004 by Ndosi *et al*. in Dar es Salaam showed that one-quarter of suicides were committed by those who were HIV positive.^29^

The highest number of hangings (55%) was recorded in the 10 to 29-year age group (Table 2 and Figure 2). This is similar to the figures in the NIMSS report. The ‘nose-dive’ pattern is seen in those who died by hanging (Figure 3). A study carried out in the Transkei region by the author in 2003 has also shown that there is an increasing incidence of suicidal deaths, and nearly two-thirds of them were among young adults younger than 30 years.^7^ Flisher et al. studied the suicide trends in South Africa from 1968 to 1990, and their study indicated that suicide among the young is increasing.^30^ Suicide rates in young people have increased during the past three decades, particularly among young males. Current research evidence suggests that the strongest risk factors for youth suicide are mental disorders in particular, affective disorders, substance use disorders and antisocial behaviours.^31^

Financial hardship was the main underlying reason identified in 87% of victims of suicide in this region.^18^ A recent (2009) study showed that the concentration of household poverty in the school community has a significant, contextual effect on adolescent suicidal behaviour.^32^ In this poverty-stricken Transkei region, the great unemployment rate (64%) along with sickness and family disintegration and excessive consumption of alcohol are the major underlying causes of suicides among young adults.^18^ Usually, suicides are uncommon in the younger age group, but the opposite was found in this study (Figure 4). Mental disorders are also predictive of suicide-related outcomes.^33^ Current research evidence suggests that the strongest risk factors for youth suicide are mental disorders.^31^ Suicide is an important cause of premature death. In the general population, most people who commit suicide have a psychiatric problem at the time. People with epilepsy are thought to be at increased risk of suicide and suicidality.^34^ A study carried out in Transkei (2004) showed a high (30 to 40%) prevalence of epilepsy between the 20- and 40-years of age groups.^35^

As shown in Figure 4, there is an early age peak in this study. The Hungarian pattern is one in which the suicide rate increases regularly with age. The Japanese pattern is one with two peaks, a smaller peak in young adulthood and a major peak in old age. The Scandinavian pattern peaks in middle age and is shaped in an inverted U-shaped curve.^36^ This could be explained by the different population profiles in these developed countries.

In economically less developed nations, the age difference in suicide rates is in the same pattern in both males and females, as it follows the upward trend (Figure 4). Women became vulnerable to suicide at a younger age in comparison to men. Dysfunctional social networks played a predominant role among suicides. Family and marital conflicts need closer social attention and timely counselling. Patients with chronic medical conditions and frequent alcohol use need effective exploration concerning suicidal ideation to avert self-annihilation.^29^

The male/female ratio has hardly changed over the 10-year period from 6.4:1 in 1993 to 6.3:1 in 2003, showing that suicide is not only a problem of males but also of females (Figure 4). It is interesting to note that the age-specific ratio is much higher among young age groups (Figure 3), namely 5.8:1 (10 to 19 years), 9:1 (20 to 29 years) and 1.2:1 (older than 70 years). This again highlights a problem of suicide among young males. In England and Wales, for example, the male to female ratio is about 3:1. Similar sex differences are reported from many countries (except China) in the developing and developed worlds.^37^ A psychiatrist in this area has shown that there is an increase in suicides among black South Africans. These reports have been supported by Mayekiso, who found that while suicide is generally unacceptable to black adolescents, a high percentage in her sample (36%) considered it an option in certain cases. She found that some of the main causes of suicides among young black adolescents in this area are an increase in parental divorce, parent-child conflict and love-relationship problems.^38^

HIV/AIDS is known to have a significant association with suicides, although in-depth studies on this topic are lacking. Earlier studies have suggested a suicide risk that is 20 to 36 times higher than in the general population.^28^ South Africa is experiencing an HIV/AIDS epidemic that has been called ‘shattering’.^39^ The rate of suicide is high in the Transkei region. Between 1996 and 2000, suicides increased parallel to the increase in mortality due to HIV/AIDS in this area of the Eastern Cape province.^40^ Although there was a debate about underestimates, the current data suggest that the rate of suicide in HIV/AIDS patients is either stabilised or decreasing in some regions of the world. The trends are primarily associated with therapeutic advances, but also others such as reduced social stigmatisation.^41^ In 2001, HIV was responsible for the death of one-third of the African population in South Africa. The calamitous advent of HIV infection has caused major falls in life expectancy.^42^ South Africa exemplifies the dichotomous economy of considerable wealth in the hands of a few, and a great deal of the population is without wealth. South Africans are exposed to trauma and violence on a daily basis.^43^

### Conclusion

In conclusion, there is an increasing incidence of suicide among teenagers and young adults in Transkei. Suicidal tendency among young adults is emerging as an important health issue. It provides important information that justifies expanded efforts to initiate and develop a programme for the screening of suicidal behaviour in this part of the country, and for the provision of urgently needed mental health services.
